# Supplementary material for: Ensa controls S-phase length by modulating Treslin levels
Source: Nat Commun. 2017 Aug 8;8:206. doi: 10.1038/s41467-017-00339-4 (PMC5547116; doi:10.1038/s41467-017-00339-4)
Supplement: Supplementary file 1 — Supplementary Information [file 41467_2017_339_MOESM1_ESM.pdf]

Title: Supplementary Information

Description: Supplementary Figures

Title: Supplementary Movie 1

Description: SC-Cell 1: Gallery of 20 z-sections at 0.7 µm steps taken each 5 min throughout DNA replication in scramble-treated cell 1. Thymidine-released Chromobody cells were followed by confocal time-lapse microscopy using a spinning disc Nikon Ti Andor CSUX1. Indicated are Bar (20 µ m), time, and appearance of early, mid and late replication origin firing.

Title: Supplementary Movie 2

Description: SC-Cell 2: Gallery of 20 z-sections at 0.7 µm steps taken each 5 min throughout DNA replication in scramble-treated cell 2. Thymidine-released Chromobody cells were followed by confocal time-lapse microscopy using a spinning disc Nikon Ti Andor CSU-X1. Indicated are Bar (20 µ m), time, and appearance of early, mid and late replication origin firing.

Title: Supplementary Movie 3

Description: SC-Cell 3: Gallery of 20 z-sections at 0.7 µm steps taken each 5 min throughout DNA replication in scramble-treated cell 3. Thymidine-released Chromobody cells were followed by confocal time-lapse microscopy using a spinning disc Nikon Ti Andor CSUX1. Indicated are Bar (20 µ m), time, and appearance of early, mid and late replication origin firing.

Title: Supplementary Movie 4

Description: SiE-Cell 1: Gallery of 20 z-sections at 0.7 µ m steps taken each 5 min throughout DNA replication in Ensa siRNA-treated cell 1. Thymidine-released Chromobody cells were followed by confocal time-lapse microscopy using a spinning disc Nikon Ti Andor CSU-X1. Indicated are Bar (20 µ m), time, and appearance of early, mid and late replication origin firing.

Title: Supplementary Movie 5

Description: SiE-Cell 2: Gallery of 20 z-sections at 0.7 µ m steps taken each 5 min throughout DNA replication in Ensa siRNA-treated cell 2. Thymidine-released Chromobody cells were followed by confocal time-lapse microscopy using a spinning disc Nikon Ti Andor CSU-X1. Indicated are Bar (20 µ m), time, and appearance of early, mid and late replication origin firing.

Title: Supplementary Movie 6

Description: SiE-Cell 3: Gallery of 20 z-sections at 0.7 μ m steps taken each 5 min throughout DNA replication in Ensa siRNA-treated cell 3. Thymidine-released Chromobody cells were followed by confocal time-lapse microscopy using a spinning disc Nikon Ti Andor CSU-X1. Indicated are Bar (20 μ m), time, and appearance of early, mid and late replication origin firing.

Title: Peer Review File

Description:

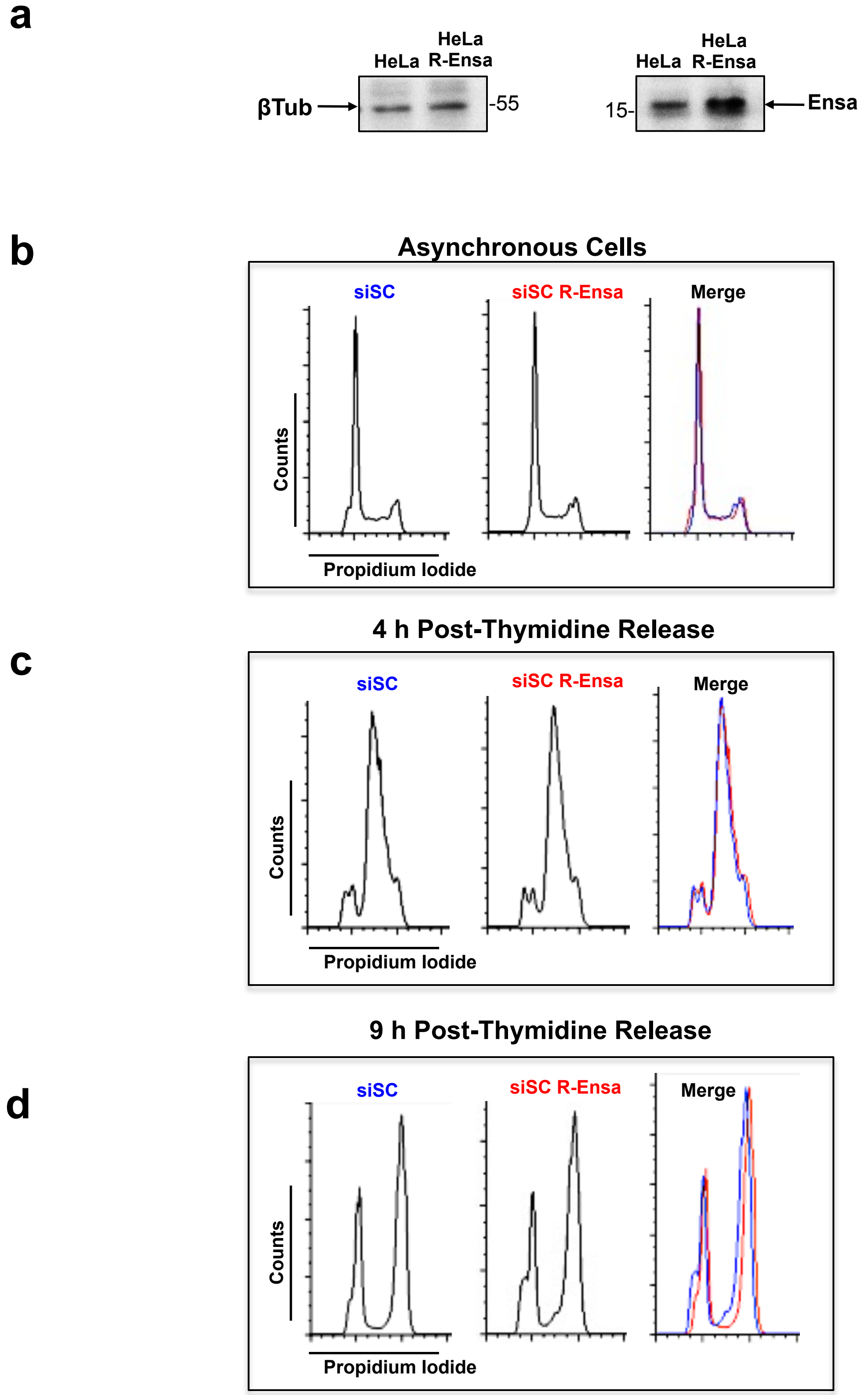

Supplementary Figure 1. *Overexpression of Ensa does not modify S-phase progression.* **(a)** Levels of Ensa protein in parental and overexpressed siRNA resistant (R-Ensa) HeLa stable cell line are shown. **(b)** Asynchronous parental and siRNA resistant Ensa overexpressing HeLa cells were transfected with scramble siRNA as for Fig. 2g and used for FACS analysis. **(c)** As for (b) except that cells were synchronised by thymidine block and recovered 4 h after thymidine release. **(d)** Cells treated as for (c) but recovered at 9 h after release.

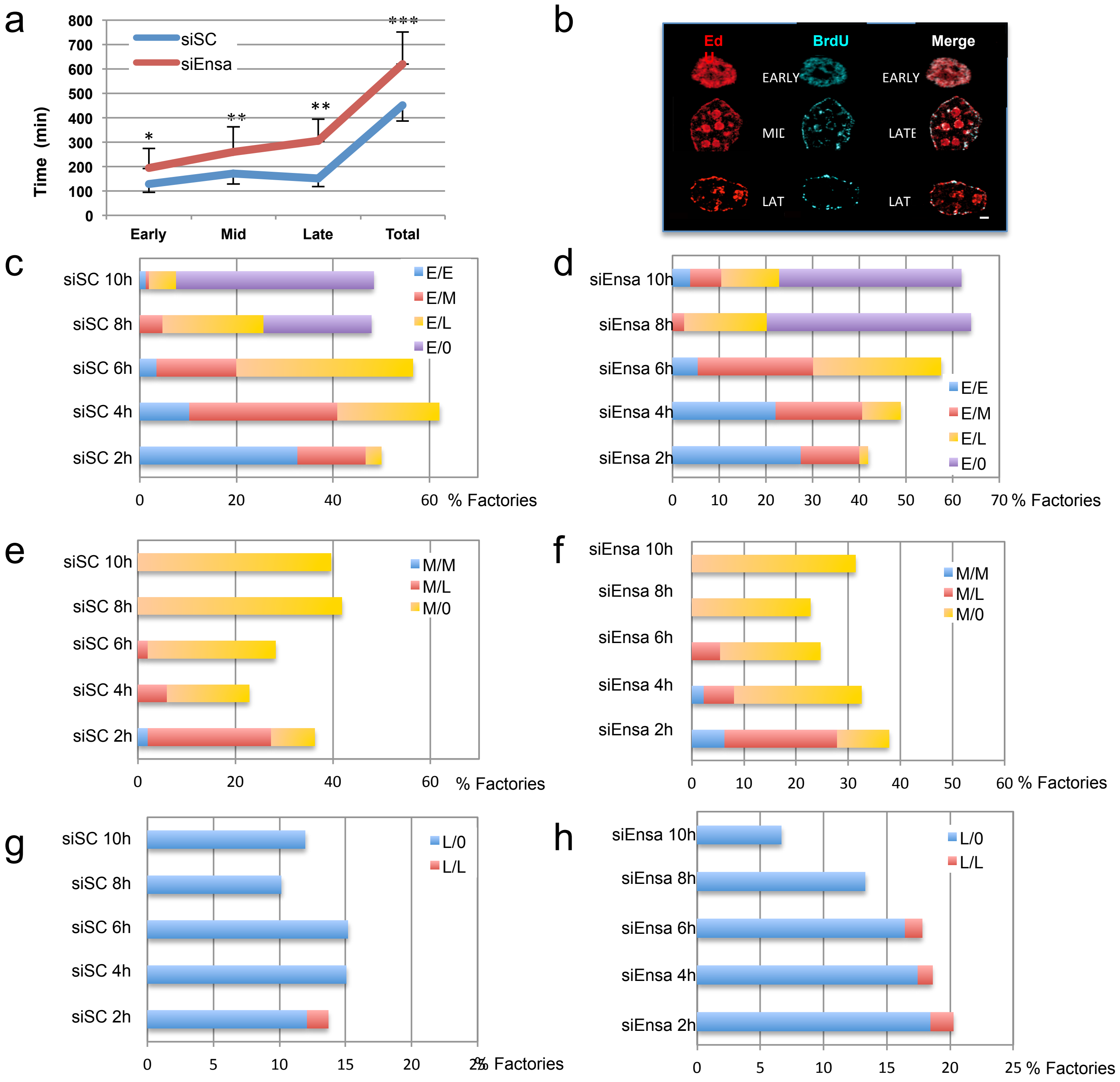

Supplementary Figure 2. *The spatiotemporal pattern of replication does not change in Ensa knocked down cells.* **(a)** Duration in minutes of early, mid or late replication factories during DNA replication in Chromobody cells treated with scramble (siSC) or Ensa siRNA (siEnsa) from Fig. 4. Results are represented as mean values +/- standard deviation. Two-tailed unpaired Student's *t* tests was performed to determine the statistical relevance. *p<0.05; **p<0.005; *** p<5 x10^{-6}. **(b)** Asynchronous Chromobody cells were labelled for 30 minutes with EdU, incubated with free medium for 2, 4, 6, 8 and 10 h and after these periods re-incubated again for 30 min with BrdU. Shown is a representative figure of early, mid and late replication origin staining after BrdU and EdU labelling. Cells were classified according to replication factory patterns in early, mid or late by both EdU and BrdU staining as shown. Bar 5 μm. **(c)** The % of siSC treated cells displaying early (E) replication factories upon EdU labelling that stayed at early (E/E), that evolved to mid (E/M), late (E/L) or that exited S phase (E/0) after BrDU labelling are shown. **(d)** As for (c) except that cells were treated with siEnsa. **(e)** siSC treated cells with mid replication factories that stayed in mid (M/M) that evolved from mid to late (M/L) or that exited S phase (M/0) are shown. **(f)** As for (e) except that cells were treated with siEnsa. **(g)** Similar representation is shown for cells starting with late replication factories (L/L and L/0) treated wiyt siSC or siEnsa **(h)**.

a

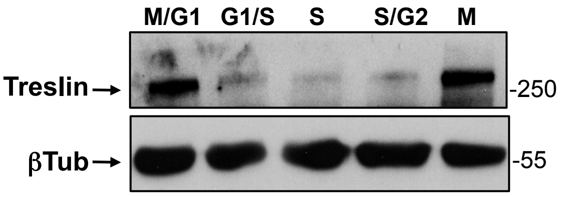

b

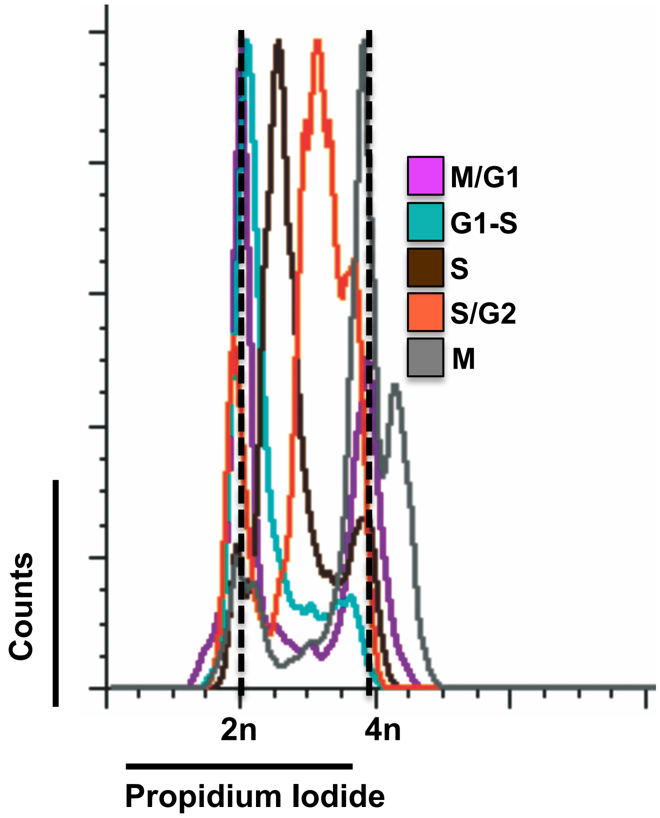

Supplementary Figure 3. *Endogenous Treslin levels during the cell cycle.* **(a)** U2OS cells were synchronized in G1 (RO-3306 5 µM block 16 h, then 5 h release), G1/S (thymidine block), S (4 h after thymidine release), G2 (7 h 30 min after thymidine release) and M (nocodazole shake off) and used for western blot with anti-Treslin antibodies. **(b)** FACS analysis of cells used in (a)

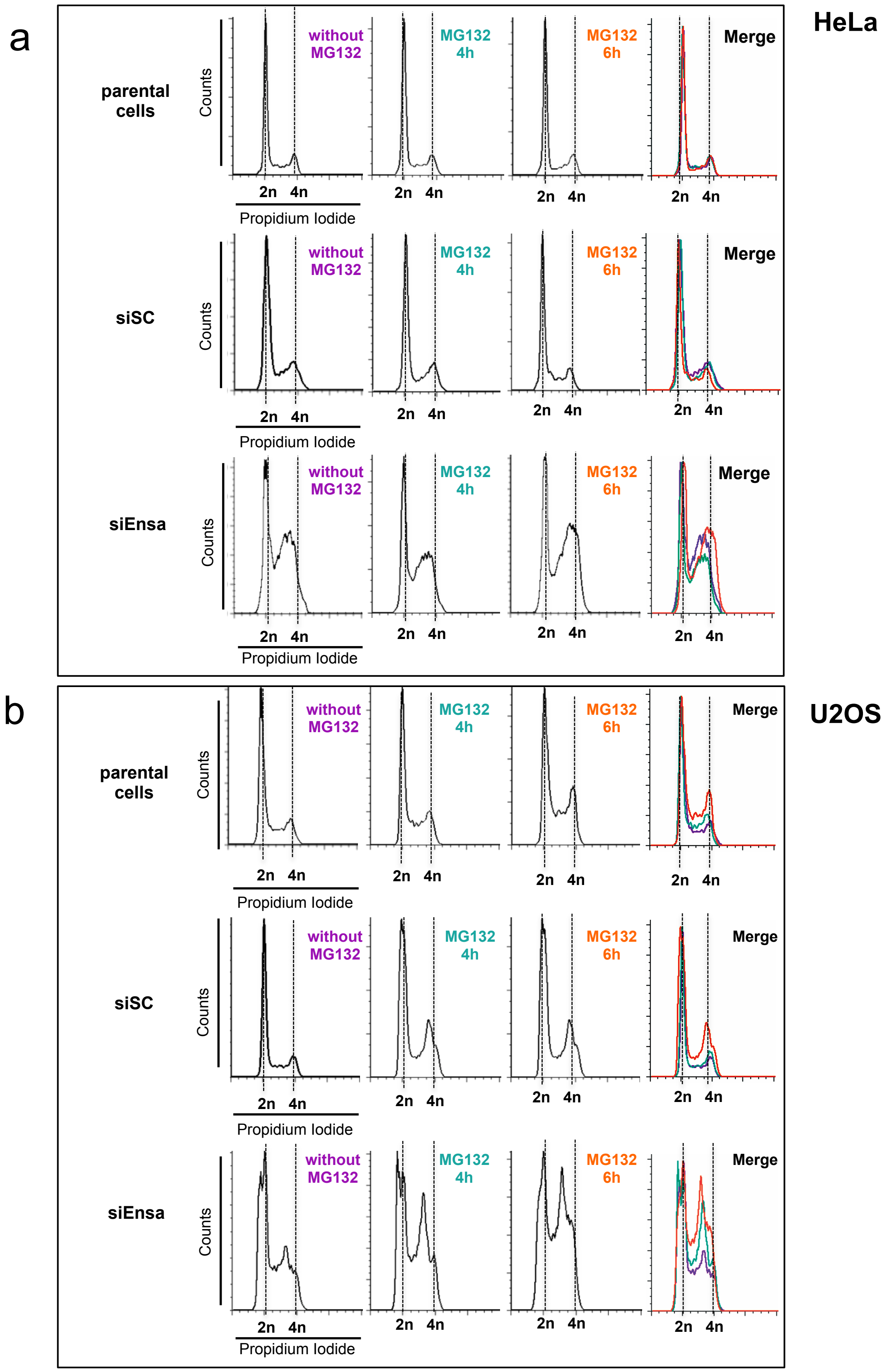

Supplementary Figure 4. *MG132 treatment does not induce different cell cycle profile in parental and siSC and siEnsa treated cells.* **(a)** Parental and siSC or siEnsa HeLa cells were treated for 4 h or 6 h with 10 µM MG132 or not and used for FACS analysis. **(b)** As for (a) except that U2OS instead of HeLa cells were used.

a

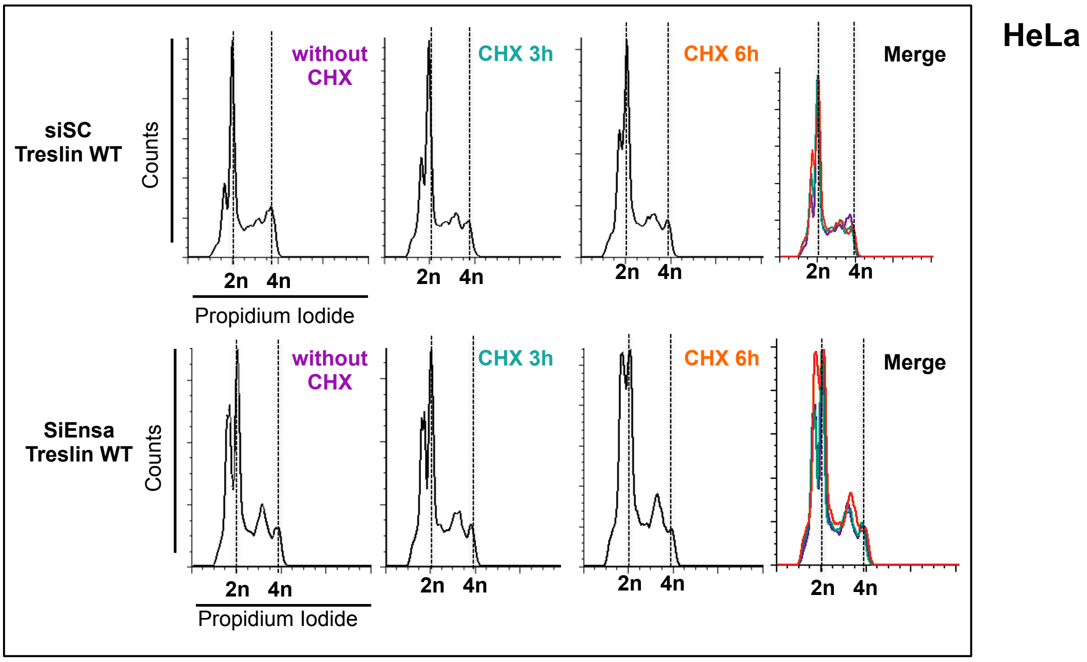

b

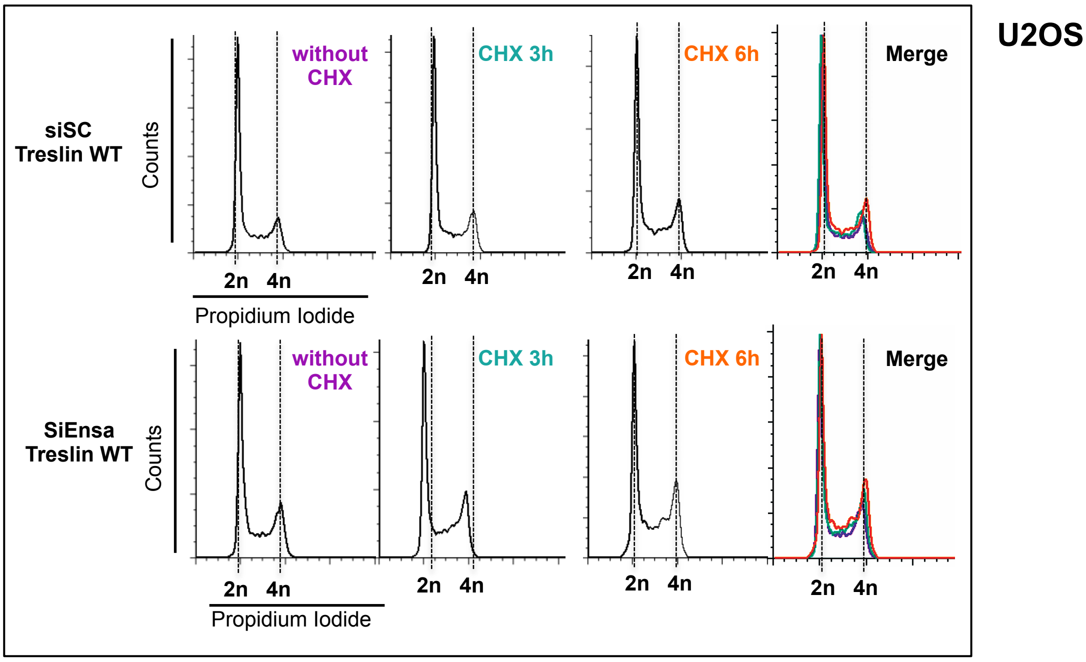

Supplementary Figure 5. *Cycloheximide does not induce different cell cycle profile in Treslin overexpressing cells treated with siSC or siEnsa.* **(a)** siSC and siEnsa treated Treslin overexpressing HeLa cells were incubated during 3 and 6 h or not with 40 μg/ml of cycloheximide and used for FACS analysis. **(b)** As for (a) except that U2OS instead of HeLa cells were used.

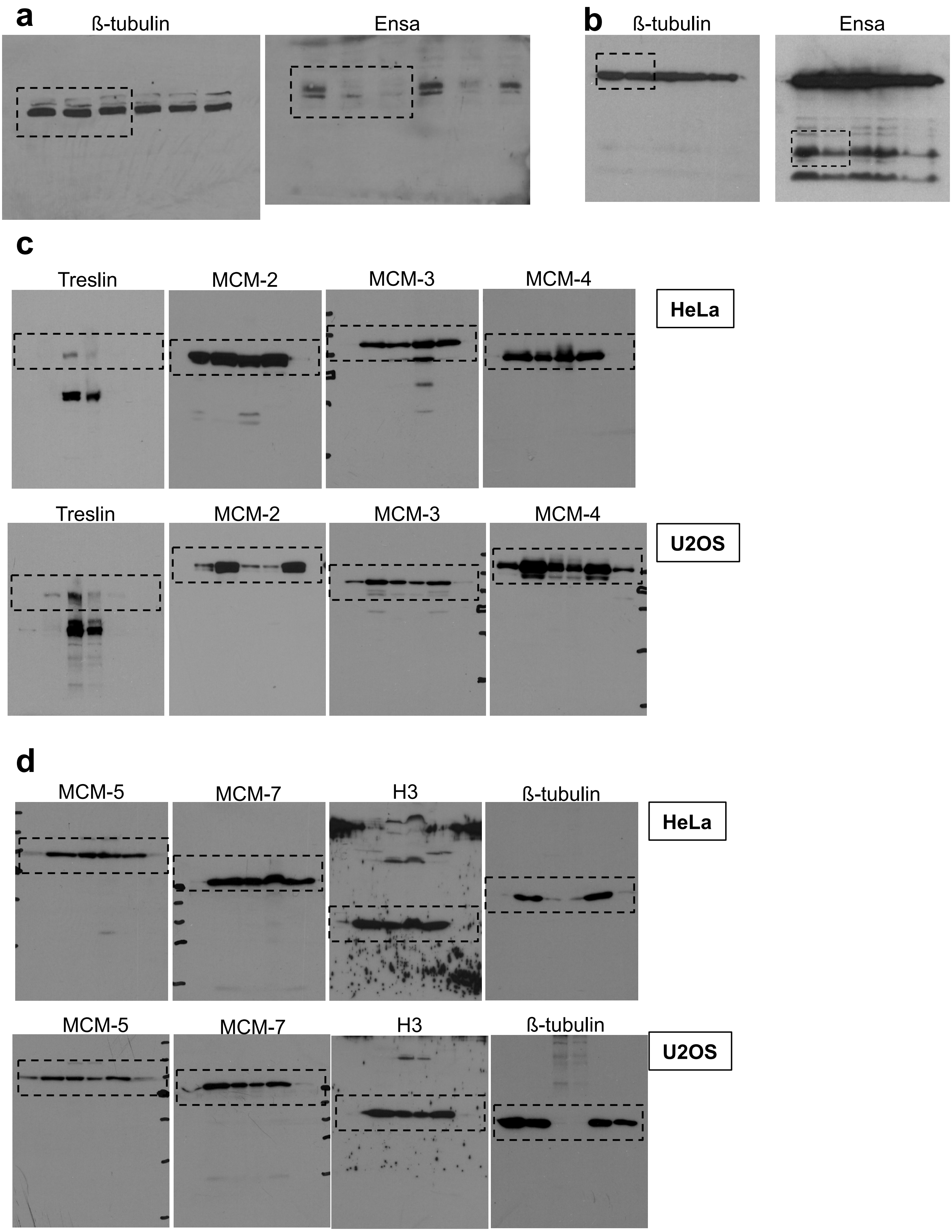

Supplementary Figure 6. *Full blot images.* **(a)** For Fig. 1a. **(b)** For Fig. 1f. **(c)** For Fig. 3g. **(d)** For Fig. 3h

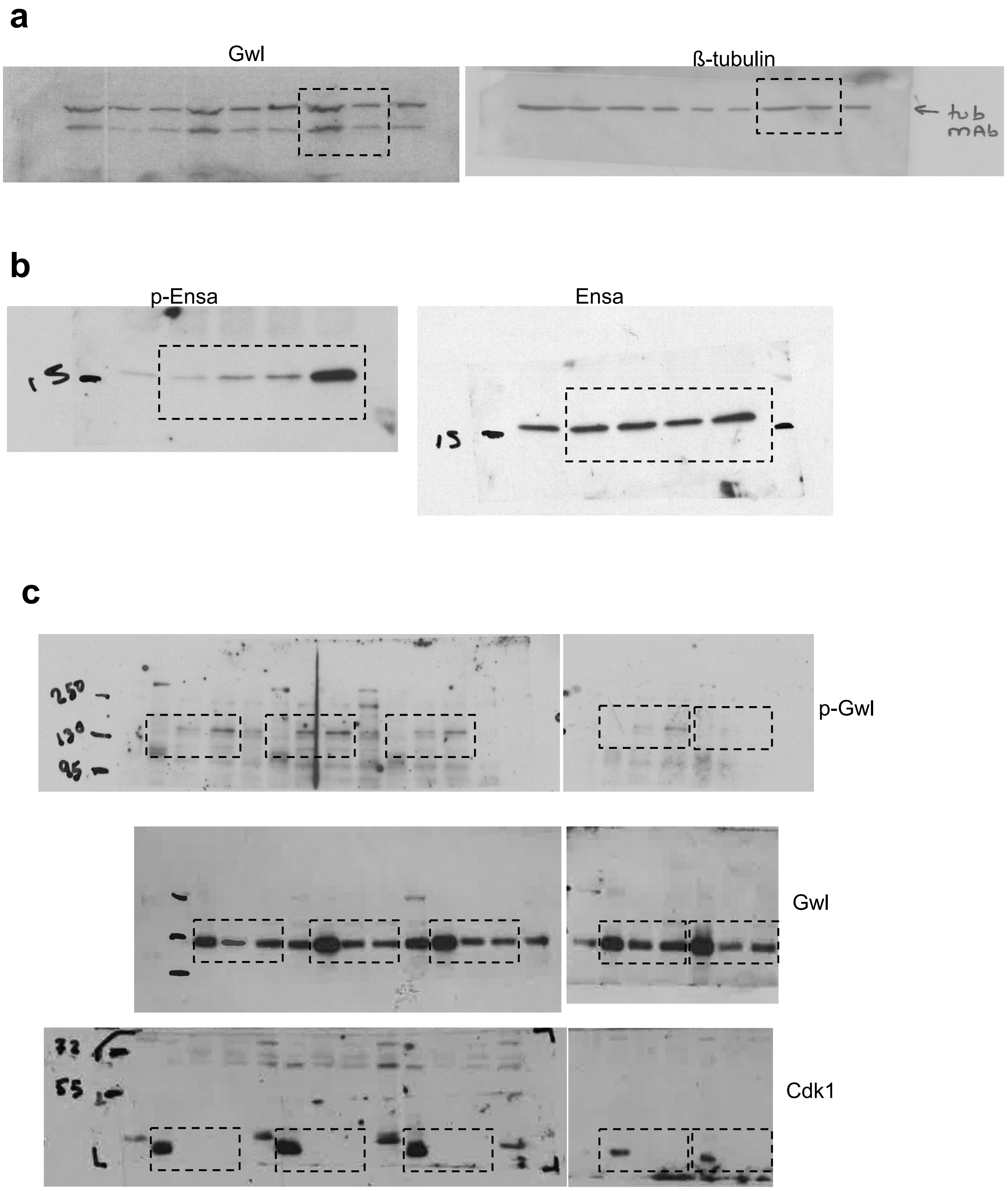

Supplementary Figure 7. *Full blot images.* **(a)** For Fig. 5a. **(b)** For Fig. 6b. **(c)** For Fig. 6c.

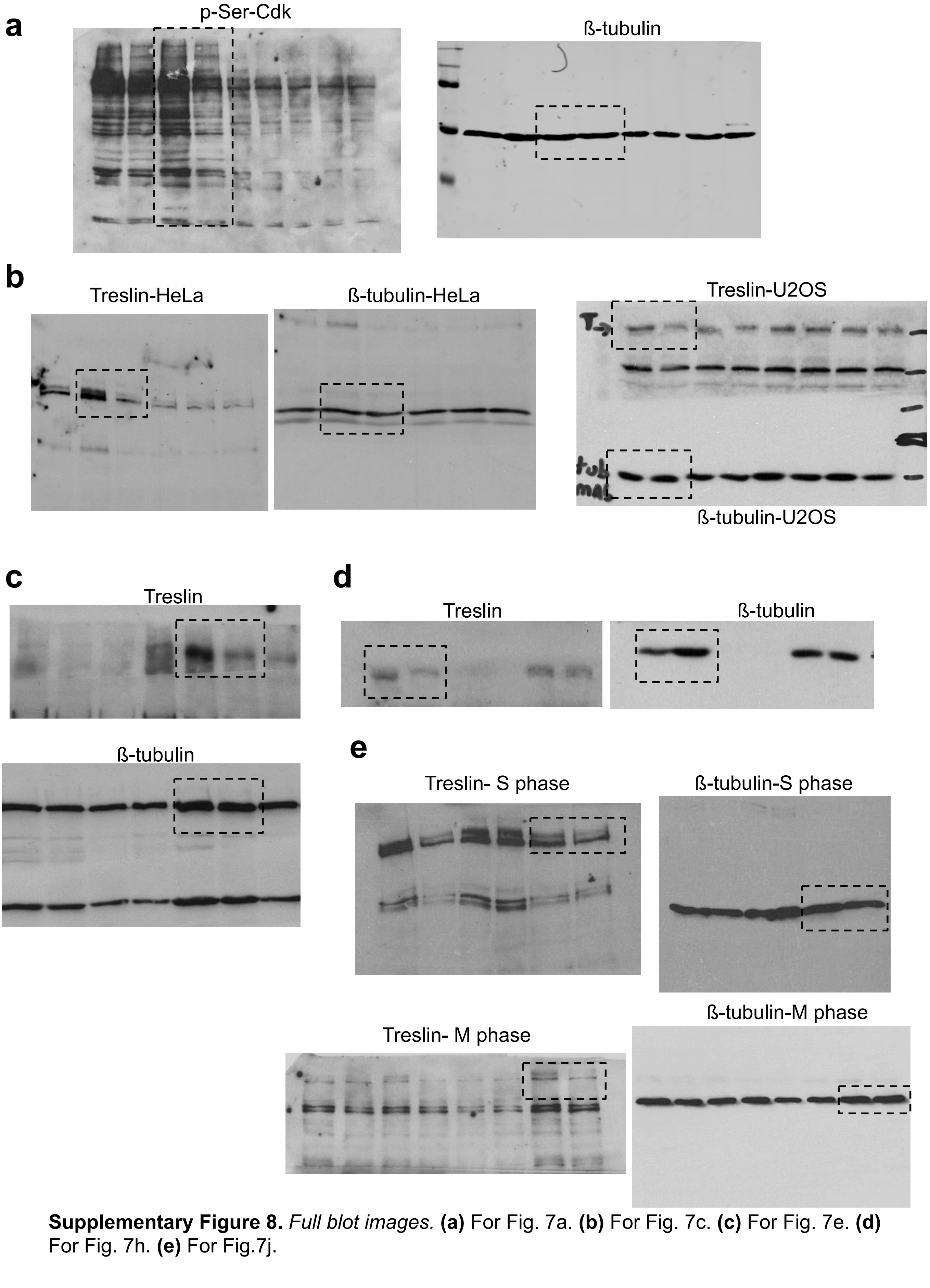

Supplementary Figure 8. *Full blot images.* **(a)** For Fig. 7a. **(b)** For Fig. 7c. **(c)** For Fig. 7e. **(d)** For Fig. 7h. **(e)** For Fig.7j.

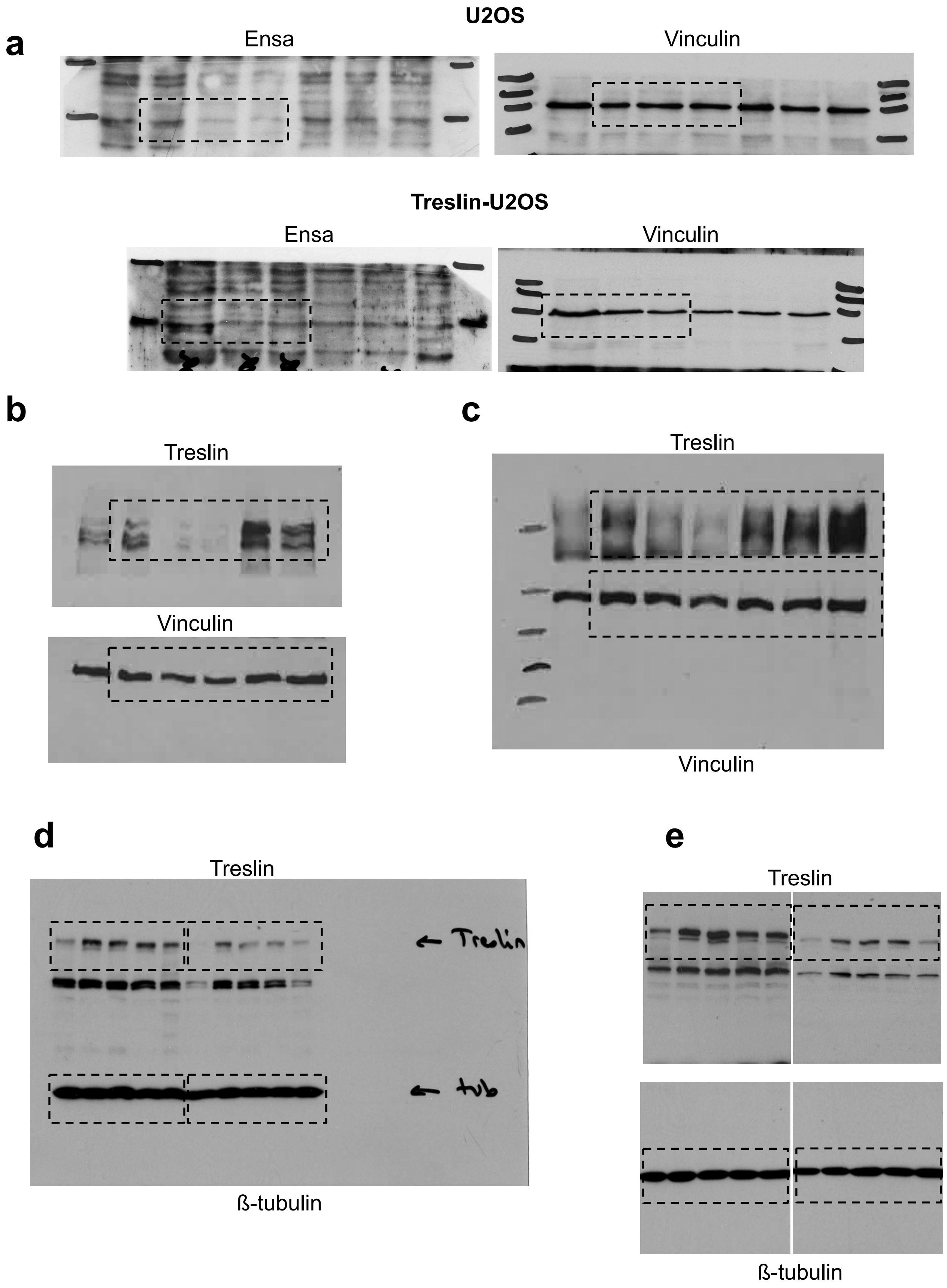

Supplementary Figure 9. *Full blot images.* **(a)** For Fig. 8a. **(b)** For Fig. 8b. **(c)** For Fig. 8c. **(d)** For Fig. 9a. **(e)** For Fig. 9b.

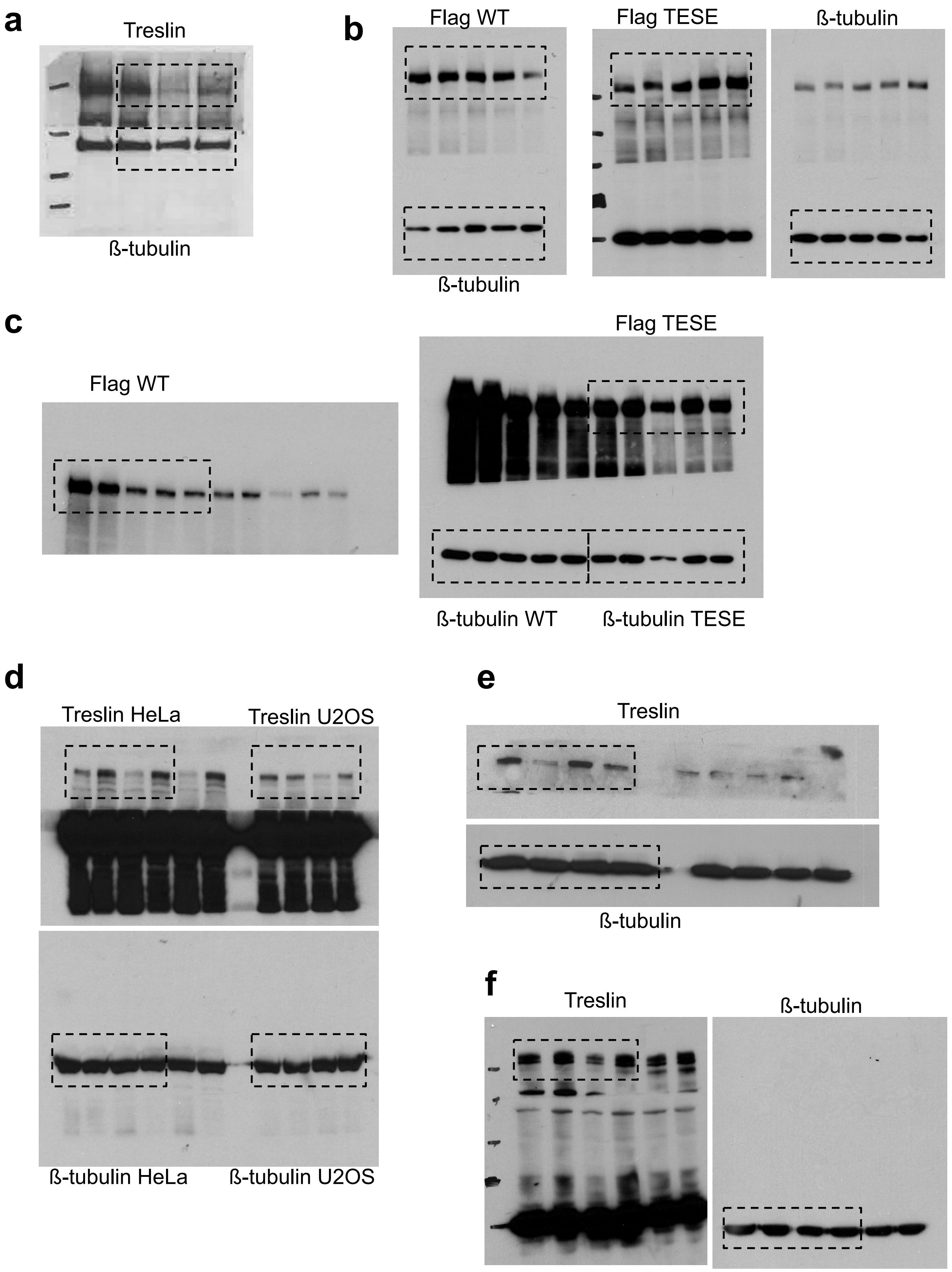

Supplementary Figure 10. *Full blot images.* **(a)** For Fig. 9c. **(b)** For Fig. 9d. **(c)** For Fig. 9e. **(d)** For Fig. 9f. **(e)** For Fig. 9g. **(f)** For Fig. 9h.

a

ß-tubulin

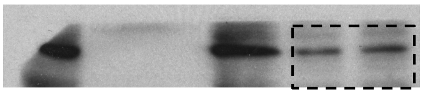

Ensa

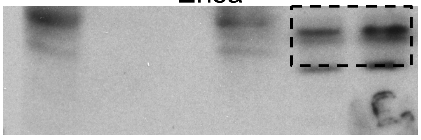

b

Treslin

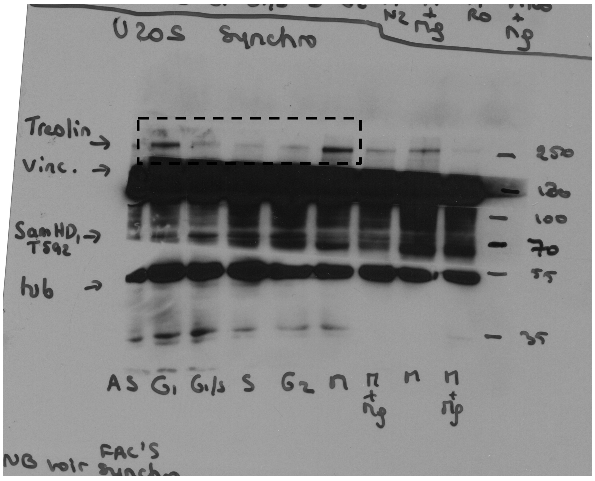

ß-tubulin

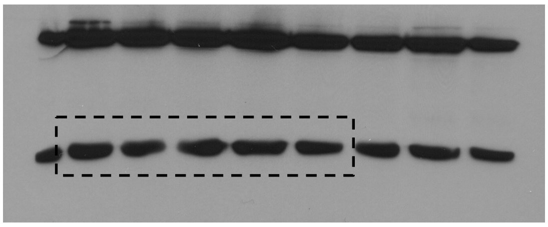

Supplementary Figure 11. *Full blot images.* **(a)** For Supplementary Fig. 1a. **(b)** For Supplementary Fig. 3a.
